# Untraceable Mobile Node Authentication in WSN

**DOI:** 10.3390/s100504410

**Published:** 2010-04-30

**Authors:** Kyusuk Han, Kwangjo Kim, Taeshik Shon

**Affiliations:** 1 KAIST, 119 Munjiro Yuseonggu, Daejeon, Korea; 2 SAMSUNG Electronics CO., LTD, Suwon, Korea

**Keywords:** wireless sensor networks, authentication, mobile node, untraceability, key distribution

## Abstract

Mobility of sensor node in Wireless Sensor Networks (WSN) brings security issues such as re-authentication and tracing the node movement. However, current security researches on WSN are insufficient to support such environments since their designs only considered the static environments. In this paper, we propose the efficient node authentication and key exchange protocol that reduces the overhead in node re-authentication and also provides untraceability of mobile nodes. Compared with previous protocols, our protocol has only a third of communication and computational overhead. We expect our protocol to be the efficient solution that increases the lifetime of sensor network.

## 1. Introduction

**Figure 1.**
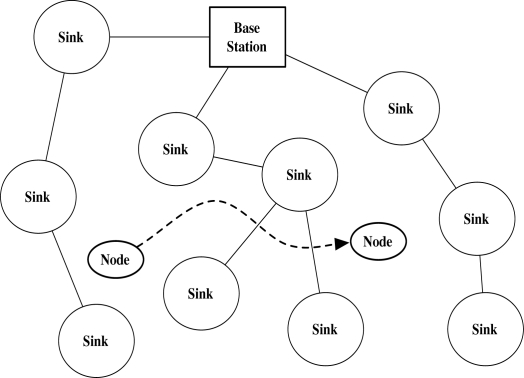
A dynamic mobile node continuously moves in the sensor networks that the static sinks established. The unbroken line denotes the static connection between sinks and the base station. The dotted line denotes the movement of the mobile node.

Wireless Sensor Network (WSN) is the network that consists of light-weight battery-powered devices with short-ranged wireless communication function. The devices have sensors that gather the environmental information. After sensing the information, the devices send the information to the networks. We define such devices as sensor node, and the core parts of the network as sinks and the base station (Figure 1).

Authenticated key distribution in WSN is one of the fundamental security problems. Employing the security protocols of other computer networks to WSN is insufficient because the light-weight devices have limited resources. Thus, the most important issues in security researches on WSN are the design of resource-efficient security protocol. Several approaches such as key pre-distribution, pairwise key agreement, group key based key agreement and hierarchical key management schemes were introduced for the efficient authenticated key distribution.

Zigbee [1] specifies the key pre-distribution method that stores the master secret between two entities for commercial application that also requires the large key storage management in scalable network. The pairwise key agreement protocols based on the random key pre-distribution that enables to share the pairwise key from the pre-distributed key pool are proposed in [2–4]. For the group key based key agreement, Zhu *et al.* [5] showed the efficient key distribution model with cluster key that enables the reduced overhead of the base station. Recently, the hierarchical key management schemes, in which the sensor nodes establish the hierarchy for the key distribution, are proposed by [6,7].

However, since the above authenticated key management protocols only considered static environments, they are not sufficient to be applied to the advanced WSN with the mobile nodes. For example, Wireless Sensor and Actor Network (WSAN) brings the concept of mobility as the extension of WSN [8,9] . It is obvious that the wireless sensor network will be the combined network of static sensor network and the mobile sensor and actor networks. In such environments, handling a large overhead from frequent node re-authentication requests due to the continuous node movements and the threats of tracing the node movement are important security issues. Thus, efficient re-authentication and untraceability are important security requirements in WSN with mobile nodes. Although Fantacci *et al.* [10] studied the possible presence of mobile node and proposed the authentication protocol supporting node mobility that does not require any sink or base station for authentication and key distribution, their model still incurs large communication overhead in node re-authentication.

Therefore, our motivation is to propose an efficient node re-authentication and key distribution model that reduces communication and computational overhead for node re-authentication. After claiming the security issues in WSN with mobile nodes, we present the insufficiency of current authentication and key distribution schemes to such environments. We then propose an efficient untraceable re-authentication and key distribution protocol that can reduce the communication overhead between a sink and the base station. Applying our protocol, a node previously authenticated by a sink can be efficiently re-authenticated with less communication and computational overhead when the node changed position and the node movement stays untraceable.

The rest of this paper is organized as follows: Section 2 briefly presents the drawbacks of previous authentication and key distribution protocols supporting mobility in WSN and identifies the security requirements. Then, We propose the efficient mobile node re-authentication protocol in Section 3, and analyze the performance and security of our protocol in Section 4. Finally, Section 5 concludes this paper.

## 2. Issues of Mobile Node Authentication in WSN

In this section, we present the security problems on node mobility in WSN and the limits of previous authentication and key agreement models. At first, we show a sensor network model with mobile nodes as in Figure 1. We define a static sensor node as Sink, a mobile node as Node, and the base station that is the core network. The node has linear movements in the network. The base station and sinks are static, which is the same as in Ibriq and Mahgoub’s model [7]. Sinks act as the gateway and link nodes to the base station, and the base station is a kind of headquarter that manages the entire networks. When a node initially joins the network, the node connects to a sink in the network and is authenticated by the sink with the help of the base station. Afterwards, the node moves and reconnects to other sink. We assume that the sink that re-authenticates the node is the neighbor sink of the sink that previously authenticated the node. The re-authentication processes frequently happen because the node continuously moves in the network.

In practical scenarios, re-authentication happens when a node lost connection to the sink or moved and connected to other sink. For the former case, the node can be easily re-authenticated to the same sink when the connection becomes available again. For the latter case, the node request the re-authentication to other sink that is closest to the previously attached sink.

### 2.1. Previous Works on the Authenticated Key Agreement in WSN

Currently, most researches on the authentication and key distribution assume WSN as a static environments. Thus, they only focused on the efficient initial authentication and key setup.

**Figure 2.**
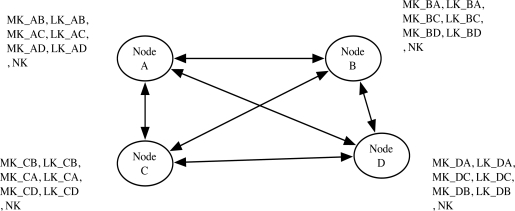
Each node has to store seven keys in order to support mobile nodes in the network with four sensor nodes under Zigbee. [1]

Commercially deployed Zigbee [1] specifies the key agreement architecture that pre-distribute keys. In their architecture, each node pre-installs their unique keys, such as the master key (MK) and the link key (LK), that are shared to other entities and the network key (NK) is shared to entire network by the manufacturer. In order to support node mobility using the unique key, each node has to contain the key as well as the number of nodes. Figure 2 shows the required keys in Zigbee. Seven keys (three MKs, three LKs, and a NK) were required for the secure communication in the network with only four nodes. Thus, deploying Zigbee in the large scale networks requires quite large storage for the key management.

In 2002, Eschenauer and Gligor [2] proposed the pairwise key agreement protocols based on the random key pre-distribution that enables sharing the pairwise key from the pre-distributed key pool. In the initial stage, each node stores *m* numbers of keys selected in a key pool. After the nodes are deployed, each node shares the key information to its neighbor nodes. When the shared keys are found, the node establishes the secure links between sinks that share the keys. After the links are established, nodes generate the pairwise key with the sink that has no shared information via the secure link. Later, Chan *et al.* [3] improved the model by generating the pairwise key from multiple numbers of shared key, and Liu and Ning [11] proposed a model in which the pairwise key is not directly distributed but derived by a bivariate polynomial. However, the networks cannot be completely connected by probabilistic methods. The probability of failure increases in the case of irregular deployment of sensor nodes or unpredictable interruptions.

Zhu *et al.* [5] introduced the group key based key agreement model that minimized threats of compromised nodes. Every node has a unique key, pairwise keys with neighbor nodes, a cluster key shared with all neighbor nodes, and the global key shared with the entire network. However, they only assumed static networks.

**Figure 3.**
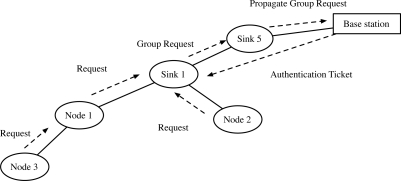
Ibriq and Mahgoub’ model [7]: The intermediate Sink 1 stores the partial key escrow table that stores the partial information of nodes. After the requests from nodes are received, Sink 1 request the authentication ticket to the base station. After receiving the ticket, Sink 1 authenticates and share keys with nodes.

In 2006, Abraham and Ramanatha [6] proposed an authentication and initial shared key establishment model in hierarchical clustered networks. In 2006, Ibriq and Mahgoub [7] proposed an efficient hierarchical key establishment model with “partial key escrow table”. Using the key escrow table, a sink can self-generate the shared key for the attached nodes. Figure 3 shows the brief model of [7]. However, any sinks have to maintain the information of every node in the table to support the node mobility.

**Figure 4.**
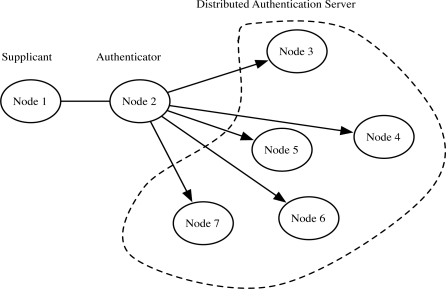
Fantacci *et al.*’s model [10]: When Node 1 request to join the network, Node 2 acts as the authenticator. Other nodes act as authentication server. In the initial setup of network, all node share the partial information of each node. When a node request to be authenticated, they gather the authentication information using secret sharing.

Fantacci *et al.* [10] proposed the distributed node authentication model that does not require the base station as the centralized authenticator. Figure 4 shows the brief model with no centralized authenticator. Every node shares the partial authentication information of each node based on Shamir’s Secret Sharing Scheme [12], which enables node mobility support. When a node requests to be authenticated to other node, the Node 2 is the authenticator, while other nodes such as Node 5 and Node 6 are distributed authentication servers. However, the issue in this model is the overhead on each node. Since the node has to participate in the authentication procedures as authenticator or an authentication server, the computational and communication overhead can increase significantly with frequent authentication requests.

Huang *et al.* [13] proposed self-organizing algorithm by using Elliptic Curve Cryptography (ECC). Once the certificates are issued to nodes, nodes can self-establish the pairwise key by exchanging the certificates with any node. Even though the public key based security architecture requires more advanced computational power and resources, efficient applications for the sensor networks will be available in near future with light weight implementation such as TinkPK [14] and TinyECC [15].

### 2.2. Drawbacks of Previous Protocols Supporting Mobile Node

#### 2.2.1. Frequent Re-authentication

Since the sensor has battery of limited power and low-end processor with short-range wireless communication, reducing communication and computational overheads is important to increase the lifetime of the sensor. However, the mobile sensor node may incur large overhead for security computation due to the frequent requests of node re-authentication. When a node connects to a sink, the sink has to authenticates the node. Afterwards, the node will connect to another sink after the movement, and the new sink has to authenticate the node again. If the node moves continuously, the authentication process will also occur repeatedly. It is obvious that the frequent re-authentication processes significantly drain the resources in battery-based sensor nodes.

**Figure 5.**
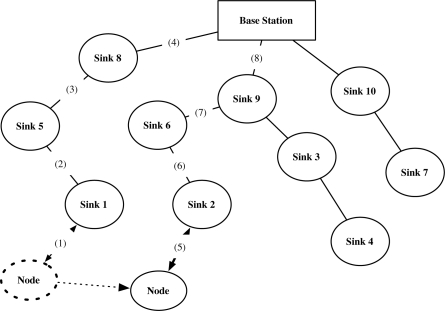
Communication pass: initial authentication (1)-(2)-(3)-(4), re-authentication (5)-(6)-(7)-(8). The unbroken line denotes the static connection, and the dotted line denotes the movement of the node.

Current authentication and key distribution protocols lacks the consideration of node mobility and are thus insufficient to be applied in such environment. Using the current protocols such as [7], the communication pass (1)-(2)-(3)-(4) is required for the initial authentication and key distribution in Figure 5. When the node moves and reconnects to sink 2, the communication pass (5)-(6)-(7)-(8) is required for authentication and key distribution, which have the similar communication overhead to the initial authentication. Such overhead will create huge problem in the environment where large numbers of nodes moves frequently. Thus, the reduction of computational and communication overheads in re-authentication are very urgent requirement for the node mobility support in the WSN.

#### 2.2.2. Tracing Node Movements

Considering the mobility of sensor nodes, the tracking of node movement is one of the possible attacks. For example, when the mobile nodes are deployed in battle fields, the tracking by enemies is of significant threat to the networks. Also, tracking node movement threats privacy. Thus, the authentication and key agreement protocols should provide the privacy of the mobile node. Current protocols do not consider the mobility of the node.

### 2.3. Security and Privacy Requirements

We define the security requirements as follows. We assume that when the node *N* communicates with a sink *S*_2_ after disconnection to the sink *S*_1_, *S*_1_ cannot receive any message between *N* and *S*_2_. *S*_2_ is one of neighbor sinks of *S*_1_.

**Re-authentication** An authenticated node *N* and *S*_2_ should be able to identify each other with less communication and computational overhead than in the initial authentication.
**Untraceability** In re-authentication of *N*, *S*_2_ only identifies that *N* was previously connected to *S*_1_, and never traces the direction of *N*.

In addition to the requirements of “re-authentication” and “untraceability”, we also define the fundamental security requirements as follows.

**Confidentiality** When *N* and *S*_1_ are operating initial authentication, nobody can know the communication packet between *N* and *S*_1_, between *S*_1_ and *BS*. For re-authentication between *N* and *S*_2_, nobody except *S*_1_ can know the communication information, while *S*_1_ out of communication range.
**Message Authentication** Any malicious adversaries should not be able to forge the communication packet.
**Key Freshness** *N* and *S* should be able to verify that the key is generated during the current session.
**Node/Sink Resiliency** Even *N*, *S*_1_ or *S*_2_ are compromised by a malicious adversary, they should not be able to affect to the entire network.

“Confidentiality”, “message authentication”, and “key freshness” are important requirements to protect against the attacks such as the replay attack or man-in-the-middle attack. “Node/Sink resiliency” is a practical threat as the sensor nodes are generally deployed in the environment out of administration.

## 3. Proposed Protocol

In this section, we propose our novel authentication and key distribution scheme that provides efficient mobile node re-authentication and untraceablity. In Section 3.1, we briefly overview the overall process of proposed protocol. In Section 3.2, we introduce the concept of “authentication ticket” that enables fast re-authentication. After that, we show our efficient node re-authentication protocol in Section 3.3.

### 3.1. Overview of Proposed Protocol

**Figure 6.**
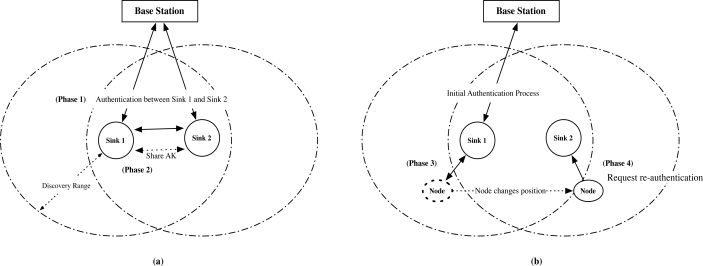
Protocol overview: Upon receiving HELLO of Sink 2 (*S*_2_), (a) Sink 1 (*S*_1_) mutually authenticates Sink 2 (Phase 1), and shares the authentication key (Phase 2). (b) Node is initially authenticated by Sink 1 (Phase 3), and requests re-authentication to Sink 2.

We briefly describe the procedure of our proposed protocol in Figure 6. Assume that there are a base station *BS*, a sink *S*_1_, a neighbor sink *S*_2_, and a mobile node N in the network. We define the neighbor sink as the sink that is in the 1 hop communication range. *S*_1_ periodically broadcasts HELLO in Phase 0. When *S*_2_ receives HELLO, *S*_2_ initiates the neighbor relationship if *S*_1_ is a newly discovered sink. After the pairwise key between *S*_1_ and *S*_2_ has been exchanged in Phase 1, *S*_1_ and *S*_2_ exchange the authentication key that is used to verify the authenticated user in Phase 2. Phase 1 and Phase 2 are only required during establishing the static sensor network. We let the establishment of the static sensor network follow any previous protocol, such as [7].

When *N* first joins the network, *N* may be connected to *S*_1_ in the network, as in Figure 6. After receiving HELLO of *S*_1_, *N* initiates the initial authentication with *S*_1_ in Phase 3. After N is authenticated *S*_1_, *N* only needs the re-authentication in Phase 4 when *N* continuously moves and request the authentication again. The authentication process in Phase 3 is only necessary when the re-authentication fails in certain case, e.g., when the neighbor sink is not available.

### 3.2. Authentication Ticket

**Figure 7.**
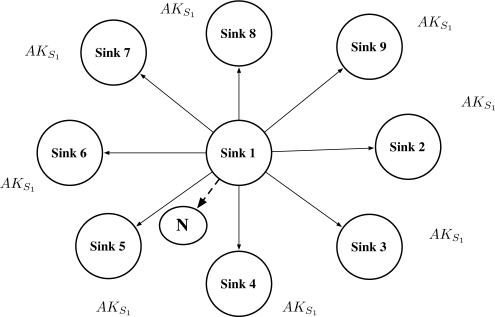
Sink 1 shares *AK_S_*__1__ to neighbor sinks. When *N* is authenticated by Sink 1, any neighbor sinks can re-authenticate *N*.

The “Authentication Ticket” is used for the node re-authentication. When a node requests authentication to a sink, the sink generates the authentication ticket and sends it to the node. The authentication ticket can be verified by the authentication key that is given to the neighbor sinks. Using the authentication ticket, the node movement is untraceable. Verification of the authentication ticket is available to neighbor sinks of the sink that issued the ticket. We adopt the idea of “cluster key” in [16] that shared to neighbor sinks. The main difference is that the cluster key in [16] is used for broadcast communication in the cluster, while the key in our protocol is used for verifying the authentication ticket. Thus, we rename the key as “authentication key” because of its different use in the protocol. Figure 7 shows that neighbor sinks of Sink 1 (*S*_1_) shares the authentication key *AK*_*S*_1__.

### 3.3. Protocol Description

The protocol consists of five phases as follows: **Phase 0** The common neighbor discovery, **Phase 1** Neighbor sink relationship set up, **Phase 2** Neighbor group authentication key share, **Phase 3** Initial node authentication, and **Phase 4** Node re-authentication.

The notations used in the protocol are defined in Table 1. Key *IK_N_* is the integrity key derived from *K_N_*, where *IK_N_* = *KDF*(*K_N_*). *KDF* is an one-way key derivation function. We can also use a hash function for *KDF*.

#### 3.3.1. Phase 0: Neighbor Discovery

**Figure 8.**
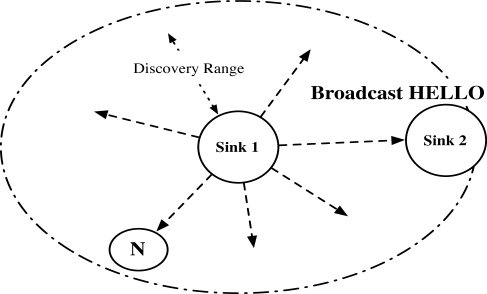
Neighbor discovery (Phase 0): sink periodically broadcasts HELLO.

A sink *S*_1_ periodically generates a random nonce *R*_0_. *S*_1_ also generates *u*_0_ = *E*_*K*_*S*_1___ {*R*_0_‖*T S*_0_} and *v*_0_ = *MAC*_*IK*_*S*_1___ (*S*_1_‖HELLO‖*u*_0_), where *TS*_0_ is time stamp. *u*_0_ and *v*_0_ are included in the HELLO message as in Figure 8. Then *S*_1_ broadcasts *u*_0_ and *v*_0_ as follows:

$$S_{1}\to \mathrm{Broadcast}:S_{1}\left\|\mathrm{HELLO}\right\|u_{0}\|v_{0}$$

Phase 0 is the periodical common procedure. When a sink receives HELLO, the sink initiates Phase 1 or Phase 2. When a node receives HELLO, the node initiates Phase 3 or Phase 4.

#### 3.3.2. Phase 1: Neighbor Sink Relationship Set Up

Assume another sink *S*_2_ receives HELLO message. *S*_2_ checks whether the sender of HELLO *S*_1_ is known or not. If *S*_2_ already knows *S*_1_, *S*_2_ discards the message. Otherwise, *S*_2_ requests to set up the neighbor relationship as follows:

**P-1.a.** *S*_2_ randomly selects *R*_1_ and generates *u*_1_ = *E*_*K*_*S*_2___ {*R*_1_‖*u*_0_}, *v*_1_ = *MAC*_*IK*_*S*_2___ (*S*_2_‖BS‖*S*_1_‖*u*_1_‖*v*_0_).

$$S_{2}\to \text{BS}:S_{2}\left\|\text{BS}\right\|S_{1}\|u_{1}\|v_{1}\|v_{0}$$

**P-1.b.** After verifying *v*_1_, BS decrypts *u*_1_ and retrieves *R*_1_ and *u*_0_. Then, BS verifies *v*_0_ and decrypts *u*_0_. Finally, BS retrieves *R*_0_ and *TS*_0_. BS generates and sends *u*_4_, *v*_4_, and *v*_3_ to *S*_2_ where, *u*_3_ = *E*_*K*_*S*_1___ {*R*_1_‖*h*(*TS*_0_)}, *v*_3_ = *MAC*_*IK*_*S*1__(BS‖*S*_1_‖*u*_3_), *u*_4_ = *E*_*K*_2__{*R*_1_‖*u*_3_} and *v*_4_ = *MAC*_*IK*_2__ (BS‖*S*_2_‖*R*_1_‖*u*_4_‖*v*_3_)

$$\mathrm{BS}\to S_{2}:\mathrm{BS}\left\|S_{2}\right\|S_{1}\|u_{4}\|v_{4}\|v_{3}$$

**P-1.c.** After verifying *v*_4_, *S*_2_ decrypts *u*_4_, and retrieves *R*_1_ and *u*_3_. *S*_2_ generates *K*_*S*_1_*S*_2__ = *KDF* (0‖*R*_0_‖*R*_1_) and *IK*_*S*_1_*S*_2__ = *KDF* (1‖*R*_0_‖*R*_1_) with *R*_0_ and *R*_1_. *K*_*S*_1_*S*_2__ is encryption key and *IK*_*S*_1_*S*_2__ is integrity key between *S*_1_ and *S*_2_. Then *S*_2_ generates *v*_5_ = *MAC*_*IK*_*S*_1_*S*_2___ (*S*_2_‖*S*_1_‖*R*_0_‖*R*_1_) and sends *u*_3_, *v*_3_, and *v*_5_ to *S*_1_.

$$S_{2}\to S_{1}:S_{2}\left\|S_{1}\right\|u_{3}\|v_{3}\|v_{5}$$

**P-1.d.** After verifying *v*_3_, *S*_1_ decrypts *u*_3_ and retrieves *R*_1_. *S*_1_ also generates *K*_*S*_1_*S*_2__ and *IK*_*S*_1_*S*_2__. Then *S*_1_ verifies *v*_5_. *S*_1_ generates *v*_6_ = *MAC*_*IK*_*S*_1_*S*_2___ (*S*_1_‖*S*_2_‖*ACK*‖*R*_0_‖*R*_1_) and sends *v*_6_ with ACK to *S*_2_.

$$S_{1}\to S_{2}:S_{1}\left\|S_{2}\right\|\mathrm{ACK}\|v_{6}$$

**P-1.e.** *S*_2_ verifies *v*_6_ and shares pairwise keys *K*_*S*_1_*S*_2__ and *IK*_*S*_1_*S*_2__.

#### 3.3.3. Phase 2: Neighbor Group Authentication Key Share

Phase 2 can be operated solely or after Phase 1 is completed. In Phase 2, *S*_1_ initiates following procedures.

**P-2.a.** *S*_1_ randomly selects two nonces *ASEED*_*S*_1__ and *R*_1_. Then *S*_1_ generates *u*_1_ = *E*_*K*_*S*_1_*S*_2___ {*ASEED*_*S*_1__‖*R*_1_} and *v*_1_ = *MAC*_*IK*_*S*_1_*S*_2___ (*S*_1_‖*S*_2_‖*u*_1_).

$$S_{1}\to S_{2}:S_{1}\left\|S_{2}\right\|u_{1}\|v_{1}$$

**P-2.b.** After verifying *v*_1_, *S*_2_ decrypts *u*_1_, and retrieves *ASEED*_*S*_1__ and *R*_1_. Then *S*_2_ generates *AK*_*S*_1__ = *KDF* (0‖*ASEED*_*S*_1__) and *AIK*_*S*_1__ = *KDF* (1‖*ASEED*_*S*_1__). *S*_2_ also generates *v*_2_ = *MAC*_*AIK*_*S*_1___ (*S*_2_‖*S*_1_‖ACK‖*AR*_1_) using *AIK*_*S*_1__.

$$S_{2}\to S_{1}:S_{2}\left\|S_{1}\right\|\text{ACK}\|v_{2}$$

**P-2.c.** *S*_1_ verifies *v*_2_.

**Figure 9.**
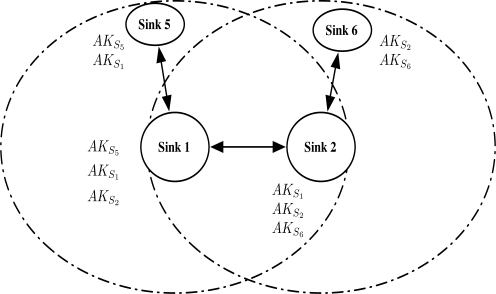
Neighbor group authentication key share (Phase 2): sinks share neighbor sink’s authentication keys.

After the Phase 2 is completed, sinks share their neighbor sink’s authentication keys as in Figure 9.

#### 3.3.4. Phase 3: Initial Node Authentication

When *N* receives HELLO that *S*_1_ broadcasts in Phase 0 and is not yet authenticated by any sink, *N* proceeds followings.

**P-3.a.** Node *N* randomly selects *R*_1_ and generates *u*_1_ = *E_K_N__* {*R*_1_‖*u*_0_‖*v*_0_} and *v*_1_ = *MAC_IK_N__* (*N*_1_‖*S*_1_‖*u*_1_).

$$N\to S_{1}:N\left\|S_{1}\right\|u_{1}\|v_{1}$$

**P-3.b.** *S*_1_ generates *v*_2_ = *MAC*_*IK*_*S*_1___ (*S*_1_‖BS‖*N*‖*u*_1_‖*v*_1_).

$$S_{1}\to \text{BS}:S_{1}\left\|\text{BS}\right\|N\|u_{1}\|v_{1}\|v_{2}$$

**P-3.c.** After verifying *v*_2_ and *v*_1_, BS decrypts *u*_1_, and retrieves *R*_0_, *u*_0_ and *v*_0_. After verifying *v*_0_, BS decrypts *u*_0_, and retrieves *R*_0_ and TS. BS checks the validity of TS and generates *u*_3_ = *E*_*K*_*N*__ {*R*_0_}, *v*_3_ = *MAC*_*IK*_*N*__ (*BS*‖*N*‖*S*_1_‖*u*_3_), *u*_4_ = *E*_*K*_*S*_1___ {*R*_1_‖*u*_3_‖*v*_3_} and *v*_4_ = *MAC*_*IK*_*S*_1___ (*BS*‖*S*_1_‖*N*‖*R*_0_‖*u*_4_).

$$\mathrm{BS}\to S_{1}:\mathrm{BS}\left\|S_{1}\right\|N\|u_{4}\|v_{4}$$

**P-3.d.** After verifying *v*_4_, *S*_1_ decrypts *u*_4_, and retrieves *R*_1_, *u*_3_ and *v*_3_. Then *S*_1_ generates *NK_N_* = *KDF* (*R*_0_‖*R*_1_). *S*_1_ generates *t* =*E*_*AK*_*S*_1___ {*TS*‖*R*_1_‖*NK_N_* } and *w* = *MAC*_*AIK*_*S*_1___ (*N*‖*t*). Next, *S*_1_ also generates *u*_5_ = *E*_*NK*_*N*__ {*TS*‖*t*‖*w*} and *v*_5_ = *MAC*_*NIK*_*N*__ (*S*_1_‖*N*‖*R*_0_‖*u*_5_).

$$S_{1}\to N:S_{1}\left\|N\right\|u_{3}\|v_{3}\|u_{5}\|v_{5}$$

**P-3.e.** After verifying *v*_3_, *N* decrypts *u*_3_ and retrieves *R*_0_. Then *N* also generates *NK_N_* and verifies *v*_5_. *N* decrypts *u*_5_ and retrieves *TS*, *t* and *w. N* generates *v*_6_ = *MAC*_*NK*_*N*__ (*N*‖*S*_1_‖*ACK*‖*R*_0_‖*R*_1_).

$$N\to S_{1}:N\left\|S_{1}\right\|\text{ACK}\|v_{6}$$

**P-3.f.** *S*_1_ verifies *v*_6_.

#### 3.3.5. Phase 4: Node Re-Authentication

When *N* receives HELLO that *S*_2_ broadcasts in Phase 0 and is previously authenticated by a sink, *N* proceeds followings.

**P-4.a.** *N* generates *v*_1_ = *MAC*_*NIK*_*N*__ (*N*‖*S*_2_‖*t*‖*w*‖*v*_0_).

$$N\to S_{2}:N\left\|S_{2}\right\|t\|w\|v_{1}$$

**P-4.b.** *S*_2_ verifies *w* and decrypts *t. S*_2_ retrieves *R*_1_, *NK_N_* and *TS*. Using *NK_N_*, *S*_2_ verifies *v*_1_. Then *S*_2_ generates *NK*′ = *KDF* (*R*_1_‖*R*_0_), also generates *t*′ = *E*_*AK*_*S*_2___ {*R*_1_‖*NK*′*_N_* } and *w*′ = *MAC*_*AIK*_*S*_2___ (*N*‖*t*′). *S*_2_ generates *v*_2_ = *h*(*NK*′*_N_*‖*R*_0_) and *u*_3_ = *E*_*NK*_*N*__ {*R*_0_‖*v*_2_‖*t*′‖*w*′}, *v*_3_ = *MAC*_*NIK*_*N*__ (*S*_2_‖*N*‖*u*_3_).

$$S_{2}\to N:S_{2}\left\|N\right\|u_{3}\|v_{3}$$

**P-4.c.** After verifying *v*_3_, *N* decrypts *u*_3_ and retrieves *R*_0_, *v*_2_, *t*′ and *w*′. Then *N* generates *NK*′*_N_* and verifies *v*_2_. *N* generates *v*_4_ = *MAC*_*NIK*′_*N*__ (*N*‖*S*_2_‖ACK‖*R*_0_‖*R*_1_).

$$N\to S_{2}:N\left\|S_{2}\right\|\text{ACK}\|v_{3}$$

**P-4.d.** After verifying *v*_4_, *S*_2_ authenticates *N*.

**Figure 10.**
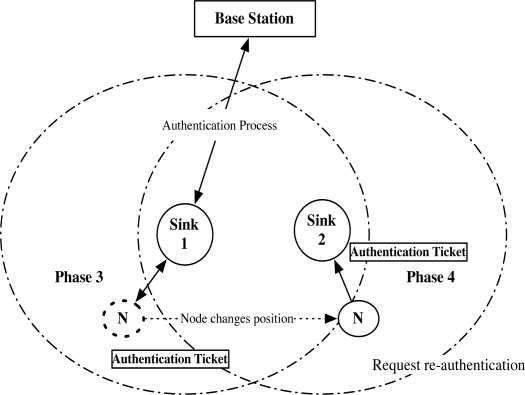
Phase 3: Node requests initial authentication to Sink 1. Phase 4: Node requests re-authentication to Sink 2

Brief procedures of Phase 3 and Phase 4 are shown in Figure 10.

## 4. Analysis

In this section, we show the performance and security analysis of our protocol. Section 4.1 shows the comparison to the previous protocols, and Section 4.2 shows the security analysis for the requirements and known attacks in WSN.

### 4.1. Performance Analysis

For the performance analysis, we compared the number of communication passes, the required message sizes, and the number of computation of the protocol. We do not count the overhead in Phase 0, since Phase 0 does not initiate the protocol. The node just ignores Phase 0 when the node receives HELLO from the sink that already authenticated the node.

#### 4.1.1. Communication Pass

We compared the required number of communication passes with Fantacci *et al.*’s model [10] and Ibriq and Mahgoub’s model [7]. The reason is that [10] considered node mobility without requiring sinks or base station in the key distribution, and [7] showed the efficient key distribution in static networks. Table 2 shows the comparison of communication passes for node re-authentication, where *n* denotes the number of nodes and *t* denotes the number of sinks. Since nodes act as the authentication server (the base station) and the authenticator (the sink), all the communications in [10] are operated among nodes.

Comparison of required number of communication pass in initial authentication is the same as the previous models. In node re-authentication, our novel protocol has much more efficiency compared with other protocols [7,10], since our protocol does not require the communication with the base station in re-authentication.

In practical application, we can deploy the network that all nodes directly connect to any sinks (*i.e.*, *n* = 1). In that case, the communication passes in our protocol are just three passes (*challenge-response-confirmation*).

#### 4.1.2. Message Size

We compared Abraham and Ramanatha’s model [6,7] for the required message size for authentication. Based on the results in [6], we approximately compared the message sizes based on the message size with MAC size as 4 bytes, the time stamp as 8 bytes, nonce as 8 bytes, and key size as 16 bytes. We also set the source and target IDs as 1 byte, respectively.

Tables 3 and 4 show the message sizes in the initial authentication and the message sizes in re-authentication with 2 hops between sink and base station, respectively. Table 3 shows that the performance for the initial authentication is similar to other protocols. In initial authentication (Phase 3), Abraham and Ramanatha’s model [6] showed the best result—30 bytes less in message sizes than our protocol. However, as Table 4 shows, our protocol achieves about a third overall message size than other protocols. Even when we increase the size of each parameter, our protocol is still much more efficient than any other protocols in node re-authentication.

**Figure 11.**
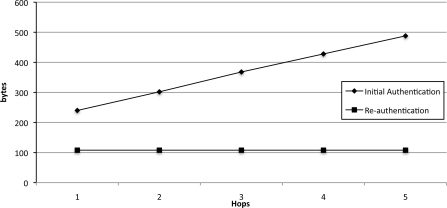
Comparison of message sizes with initial authentication and re-authentication per hop distance from sink to the base station increases.

**Figure 12.**
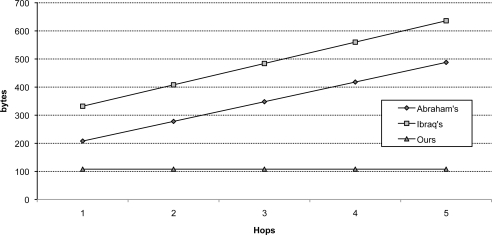
Comparison of message sizes with [6] and [7] per hop distance between a sink and a base station

For the comparison in multi-hop environments, Figures 11 and 12 show the message sizes of initial authentication (Phase 3) and re-authentication (Phase 4) in our protocol and the comparison with other protocols, respectively. When the hop distances between the sinks to which the node is attached and the base station increase, the required message size and the communication pass also increase.

#### 4.1.3. Computation

Now, we compare the computational overhead of initial authentication (Phase 3) and re-authentication (Phase 4). In total, 10 times of encryption/decryption and 14 times of MAC generation/verification are required for initial authentication, while 4 times of encryption/decryption and 10 times of MAC generation/verification are required for re-authentication. For node specific operation, 3 times of encryption/decryption for initial authentication, 1 time of encryption/decryption are required. Both cases require 4 times of MAC generation/verification. Since the computation of MAC does not have significant overhead, comparing the computation of encryption and decryption, our computation is 2–3 times more efficient. The comparison of computation is shown in Table 5. We do not measure the computation time of each operation that depends on the encryption and hash algorithms in this paper. Note that we can apply TinySEC [17] and TinyHash [18] for the implementation.

### 4.2. Security Analysis

We show the security analysis of our protocol that holds the requirements defined in Section 2.3. “re-authentication”, “untraceability”, “confidentiality”, “message integrity”, “key freshness”, and “node/sink resiliency”. Then, we analyze the security of our protocol against known attacks.

#### 4.2.1. Re-Authentication

After a node *N* is initially authenticated by a sink *S*_1_ in phase 3, the node receives the authentication ticket (*t*, *w*) and *v*_1_, where *t* = *E*_*AK*_*S*_1___ {*TS*‖*R*_1_‖*NK_N_*}, *w* = *MAC*_*AIK*_*S*_1___ (*N*‖*t*) and *v*_1_ = *MAC_NIK_N__* (*N*‖*S*_2_‖*t*‖*w*‖*v*_0_). When *N* moves and requests re-authentication to the neighbor sink *S*_2_, *S*_2_ can verifies (*t*, *w*) since the authentication key of *S*_1_, *AK_S_*__1__ is shared to *S*_2_ *N* can authenticates *S*_2_ with *u*_3_ and *v*_3_ with *NK_N_* . Finally, *S*_2_ authenticates *N* after verification of *v*_4_. In the re-authentication phase, the base station is not involved.

#### 4.2.2. Untraceability

**Figure 13.**
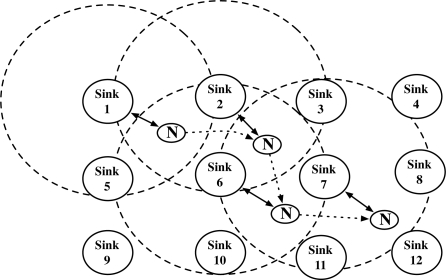
When *N* move in the networks, sinks re-authenticate *N* without knowing the node’s direction.

A sink *S*_1_ issues the authentication ticket (*t*, *w*) to a node *N*. However, *S*_1_ does not know the next move of *N. N* can be re-authenticated by any neighbor sinks of *S*_1_. For the re-authenticated sink *S*_2_, *S*_2_ only knows that *N* was previously authenticated by *S*_1_, but never knows the direction *N* ahead. Sinks only know *N* was previously authenticated by neighbor sinks, but never predict *N*’s next direction as in Figure 13.

#### 4.2.3. Confidentiality

Any sinks and nodes pre-share secret keys only with the base station. For the Neighbor discovery phase, the neighbor discovery message is encrypted using *K_S_* that is only shared between a sink and the base station. For setting up the neighbor group and node authentication, the adversary requires shared secret key to know the information. For the node re-authentication, the responses *u*_3_ and *v*_3_ are encrypted using *NK_N_* that is known to *S*_1_. However, we assume that the re-authentication happens, where *S*_1_ cannot involve in the communication from out-of-reach.

#### 4.2.4. Message Authentication

In our protocol, every packet is protected by 4 bytes MAC. The outside adversary should be able to forge the message to succeed in the attack. The security of the MAC depends on the security of the hash function. The recommended MAC size in [17] is 4 bytes for practical application, since only 40 forgery attempts per second are available on a 19.2 kb/s channel while 2^31^ trials are required for successful forgery. However, the performance of communication channel is increasing, and the size of MAC should be increased in future applications. Recently the efficient implementation of hash functions is introduced in [18]. Thus, our protocol is secure against the man-in-the-middle attack, as the adversary has no efficient way to forge MAC even when the part of the network is compromised by the attacker.

#### 4.2.5. Key Freshness

In Phase 0, the sink *S*_1_ periodically generates random nonce *R*_0_. Thus, *S*_1_ can verify that the requests of authentication are from the directly linked sinks or nodes. In Phase 1, two entities generate the random nonces whose freshness can be checked by both entities. In Phase 2, *S*_1_ also generates random nonce *R*_1_ for the freshness check. In Phase 3 and 4, the node also generates random nonce *R*_1_ to check the freshness.

#### 4.2.6. Node/Sink Resiliency

We can define two kinds of threat of sink capture: the sink missing case and the compromised sink case. When a sink *S*_1_ is just missing, the node will lose the connection *S*_1_ and find other sink such as *S*_2_. Thus, we only need to consider the compromised sink case.

When the sink is compromised, we can assume that the keys in the sink are leaked. However, even if the group authentication key is leaked, only will the neighbor sinks be affected. The compromised sink can self-attach the fake nodes that will request re-authentication without initial authentication. For this case, we add *h*(*K_N_*‖*R*_1_) in the authentication ticket that is sent to the sink when the node requests re-authentication. For suspicious nodes, the sink can check if the node is genuine with help of the base station. Also, we need to define the security policy for the extreme abnormality in deploying sensor network application. When the node is compromised, we can define that the compromised node may try to know the information of the sinks or impersonate other nodes. However, the compromised node will fail in both cases, since the node does not share any information in the protocol. Thus, our protocol has node and sink resiliency, and is practically secure against selective forwarding and acknowledgement spoofing.

#### 4.2.7. Security Against Known Attacks

We analyze the security of our protocol against the attacks identified in [19]. Since the static parts in the networks could follow the previous models such as [7], we only focus on the security of node re-authentication in this section.

The sinkhole attack against our protocol fails without knowing the keys. An adversary *A* may capture the authentication ticket (*t*, *w*) that *N* initially sent to *S*_2_, and *A* send (*t*, *w*) to *S*_2_ or other sink *S*_5_ that is also a neighbor sink of *S*_1_. However, *A* fails in such attack without knowing *AK_S_*__1__. Wormhole attack on our protocol fails since the adversary cannot send the confirmation message. Spoofed, altered or replayed routing information attack also fail without knowing the encrypted nonce in our protocol. To succeed in the replay attack, the adversary has to be able to re-use the intercepted packet. We do not consider relaying through the attackers as successful attack. Sybil attack also fails from verification of identity of nodes through sinks and the base station. As for HELLO flood attacks, we can apply the global key shared to all entities in the network that many researches such as [7,16] used for the efficient message broadcast and DoS attack protection.

## 5. Conclusions

Node mobility is one of the emerging issues in WSN that needs to be adequately addressed. In this paper, we outlined the drawbacks of previous authentication protocols supporting mobile nodes in WSN, and identified the following requirements: efficient node re-authentication and untraceability. We then proposed our novel efficient node authentication and key distribution protocol that provides re-authentication and untraceability. Also, we analyzed our protocol by comparing it with the previous protocols. Our protocol requires only three passes of communication with one third of communication message sizes compared with previous protocols in node re-authentication. The computational overhead of node re-authentication of a single mobile node achieves about 2–3 times more efficiency than that of initial node authentication. It is obvious that deploying our protocol in the environment with large numbers of mobile nodes will achieve much higher cost efficiency than any previous methods. Our future plan is to gain the energy efficiency of sensor network in the initial authentication process of our protocol. Thus, We expect that our proposed protocol will be the efficient security solution supporting mobile nodes in WSN.

## Figures and Tables

**Table 1.** Notation

Term	Description	Term	Description
BS	Base Station	*E_t_*{*m*}	Encrypt arbitrary message *m* using *t*
*h*{*m*}	Hash arbitrary message *m*	*MAC_t_*(*m*)	Message Authentication Code using *t*
TS	Time stamp	*K_N_*	Pre-shared key between *N* and *BS*
*IK_N_*	IK derived from *K_N_*	*K_S_*	Pre-shared key between *S* and *BS*
*IK_S_*	IK derived from *K_S_*	*SK*	Shared session key between sinks
*SIK*	IK derived from *SK*	*AK_S_*	Group Authentication Key of Sink
*AIK_S_*	IK derived from *AK_S_*	*NK*	Shared session key between *S* and *N*
*NIK*	IK derived from *NK*	IK	Integrity Key

**Table 2.** Comparison of required communication pass for re-authentication.

	Fantacci *et al.*’s Model [10]	Ibriq and Mahgoub’s model [7]	Proposed
Node	2	2*n*	2*n*
Sink	2*t* + 1	2*t*	1
Base station	−	2	−

**Table 3.** Comparison of required message size for initial authentication (bytes).

	Abraham’s model [6]	Ibriq and Mahgoub’s model [7]	Proposed
Node to Sink	46	68	56
Sink to Sink	70	76	62
Sink to Base station	70	76	66
Base station to Node	92	188	180
Total message size	278	408	302

**Table 4.** Comparison of required message size for re-authentication (bytes).

	Abraham’s model [6]	Ibriq and Mahgoub’s model [7]	Proposed
Node to Sink	46	68	44
Sink to Sink	70	76	-
Sink to Base station	70	76	-
Base station to Node	92	188	64
Total message size	278	408	108

**Table 5.** Comparison of computation between initial authentication and re-authentication (times).

	Initial Authentication	Re-authentication.
Encryption/Decryption in Total	10	4
Encryption/Decryption by Node	3	1
MAC Generation/Verification in Total	14	10
MAC Generation/Verfication by Node	4	4
